# Prediction of malignant esophageal fistula in esophageal cancer using a radiomics-clinical nomogram

**DOI:** 10.1186/s40001-024-01746-2

**Published:** 2024-04-04

**Authors:** Chao Zhu, Wenju Sun, Cunhai Chen, Qingtao Qiu, Shuai Wang, Yang Song, Xuezhen Ma

**Affiliations:** 1 School of Basic Medicine, Qingdao University, Qingdao, 266000 China; 2 Department of Oncology, Affiliated Qingdao Central Hospital of Qingdao University, Qingdao Cancer Hospital, Qingdao, 266042 China; 3 Department of Radiation Oncology, Shandong Cancer Hospital, Jinan, 250117 China; 4 Department of Radiation Oncology, Affiliated Hospital of Weifang Medical University, Weifang, 261000 China

## Abstract

**Background:**

Malignant esophageal fistula (MEF), which occurs in 5% to 15% of esophageal cancer (EC) patients, has a poor prognosis. Accurate identification of esophageal cancer patients at high risk of MEF is challenging. The goal of this study was to build and validate a model to predict the occurrence of esophageal fistula in EC patients.

**Methods:**

This study retrospectively enrolled 122 esophageal cancer patients treated by chemotherapy or chemoradiotherapy (53 with fistula, 69 without), and all patients were randomly assigned to a training (*n* = 86) and a validation (*n* = 36) cohort. Radiomic features were extracted from pre-treatment CTs, clinically predictors were identified by logistic regression analysis. Lasso regression model was used for feature selection, and radiomics signature building. Multivariable logistic regression analysis was used to develop the clinical nomogram, radiomics-clinical nomogram and radiomics prediction model. The models were validated and compared by discrimination, calibration, reclassification, and clinical benefit.

**Results:**

The radiomic signature consisting of ten selected features, was significantly associated with esophageal fistula (*P* = 0.001). Radiomics-clinical nomogram was created by two predictors including radiomics signature and stenosis, which was identified by logistic regression analysis. The model showed good discrimination with an AUC = 0.782 (95% CI 0.684–0.8796) in the training set and 0.867 (95% CI 0.7461–0.987) in the validation set, with an AIC = 101.1, and good calibration. When compared to the clinical prediction model, the radiomics-clinical nomogram improved NRI by 0.236 (95% CI 0.153, 0.614) and IDI by 0.125 (95% CI 0.040, 0.210), *P* = 0.004.

**Conclusion:**

We developed and validated the first radiomics-clinical nomogram for malignant esophageal fistula, which could assist clinicians in identifying patients at high risk of MEF.

## Introduction

Malignant esophageal fistula (MEF) is a serious complication of advanced esophageal cancer and is defined as a fistula caused by malignancies [1]. The two most common types are esophageal mediastinal fistula and esophageal respiratory fistula. Esophageal aortic fistula was rarely reported, because these patients often die of sudden massive hemorrhage and cannot be diagnosed before death [2]. In untreated EC patients, the incidence of esophageal fistula ranges from 5 to 15% [3], while radiotherapy patients have a range of 5.6% to 33% [4–7]. MEF has a very poor prognosis; patients frequently die within 3 months due to nutritional failure, pneumonia, mediastinal abscess or large vascular injury [8]. Patients with MEF must be treated with a nutrition tube, gastrostomy, or esophageal stent to prevent digestive fluid leakage into the trachea or mediastinum, all of which significantly reduce quality of life. Therefore, early identification of patients at risk of MEF enables early intervention, which improves patient outcomes.

However, few studies on MEF risk factors have been conducted, the majority of which were retrospective analyses of small samples, and only a few risk factors had been confirmed. T_4_ stage (invasion of adjacent organs) had been a known risk factor for MEF. Because the esophagus is a thin-walled organ with a thickness of less than 5 mm, tumors can grow through the entire thickness of the esophageal wall, which is the primary cause of MEF [3]. It is reported that the incidence of esophageal fistula is 10–22% in T_4_ patients with radical concurrent chemoradiotherapy, which is significantly higher than in non-T_4_ patients [9, 10]. In a retrospective study, ECOG PS, BMI, T_4_, N_2/3_ and re-radiotherapy were identified as independent factors, with a C-index of a nomogram incorporating the factors of 0.764 in an external validation cohort [11]. Another retrospective study revealed quantitative pretreatment CT analysis has excellent performance for predicting fistula formation in esophageal cancer patients [12].

Recently, radiomics has proven to be a useful tool for identifying the biological behavior of tumors [13]. Its central concept is to convert images into digital information that can be mined, extracted, and analyzed, as well as to predict target associations and build model [14, 15]. Iwashita et al. reported CT radiomics and clinical data might help determine survival outcomes in patients with esophageal cancer treated with radical radiotherapy [16]. Yang et al. found that radiomics can be used for chemoradiation outcome prediction in esophageal cancer [17]. Furthermore, some research has demonstrated that radiomics of tumor target areas can be utilized to predict tumor stage, differentiation, and pathological type [18, 19]. The present study attempt to develop a joint prediction nomogram by combining clinical factors and pre-treatment CT radiomics to accurately predict MEF. It will help clinicians identify EC patients at high risk of having an esophageal fistula.

## Materials and methods

### Patients

The Shandong Cancer Hospital ethics committee approved this retrospective study (Approval No. 2021003193), and informed consent was waived.

Inclusion criteria were: (a) Esophageal carcinoma proved by pathology; (b) Chest enhanced CT examination before treatment; (c) Patients treated with chemotherapy or chemoradiotherapy. Exclusion criteria: (a) Esophageal fistula appeared prior to treatment; (b) Anastomotic fistula or other medical injuries caused esophageal fistula.

A total of 122 patients treated at Shandong Cancer Hospital from October 2018 to September 2020 were included in the study. Fifty-three patients with MEF were identified, while 69 patients did not have a MEF within their survival period.

Malignant esophageal fistula must be confirmed by endoscopy or esophagography. All cases were divided into a training set and a validation set in a 7:3 ratio using a random algorithm.

### Data collection and variable definition

Clinical data such as gender, age, tumor location, TNM stage, radiotherapy, chemoradiotherapy, chemotherapy regimen, and stenosis were obtained using the hospital information system (HIS). To obtain enhanced chest CT images, the picture archiving and communication system (PACS) was used. The image format was digital imaging and communications in medicine (DICOM).

Stenosis was defined as the inability to pass an endoscope through and/or significantly impairing eating (semi-fluid or fluid diet). The chemotherapy regimens mentioned in the article were first-line treatments.

### Clinical predictors and clinical nomogram

Logistic univariate and multivariate regression analyses were used to identify clinical independent predictors and create a clinical nomogram using the variables with *P* < 0.25 in multivariate analyses.

### Tumor segmentation

The region of interest (ROI) was defined as primary tumors, which included lesions with esophageal wall thickening > 5 mm or lumen occlusion diameter > 10 mm but did not include intraluminal gas or oral contrast agent. A radiation oncologist with 10 years of experience manually delineated the ROIs, which were then checked by a radiologist. Both doctors were unaware of the patient’s clinical information. 3D Slicer (version: 4.10.2), an open source software platform for medical image processing and visualization, was used to perform all delineation tasks.

### Feature extraction and selection

Radiomic features were extracted automatically from each contoured ROI using Pyradiosity, an open source Python package available at https://pyradiomics.readthedocs.io/en/latest/.

The Lasso-logistic regression algorithm was used to screen radiomic features related to the presence of MEF. The Pearson correlation test was used to examine the multicollinearity.

### Radiomics model construction

Based on the screened features, the logistic regression algorithm generated the radiomics model.

### Radiomics-clinical model construction

**Fig. 1:**
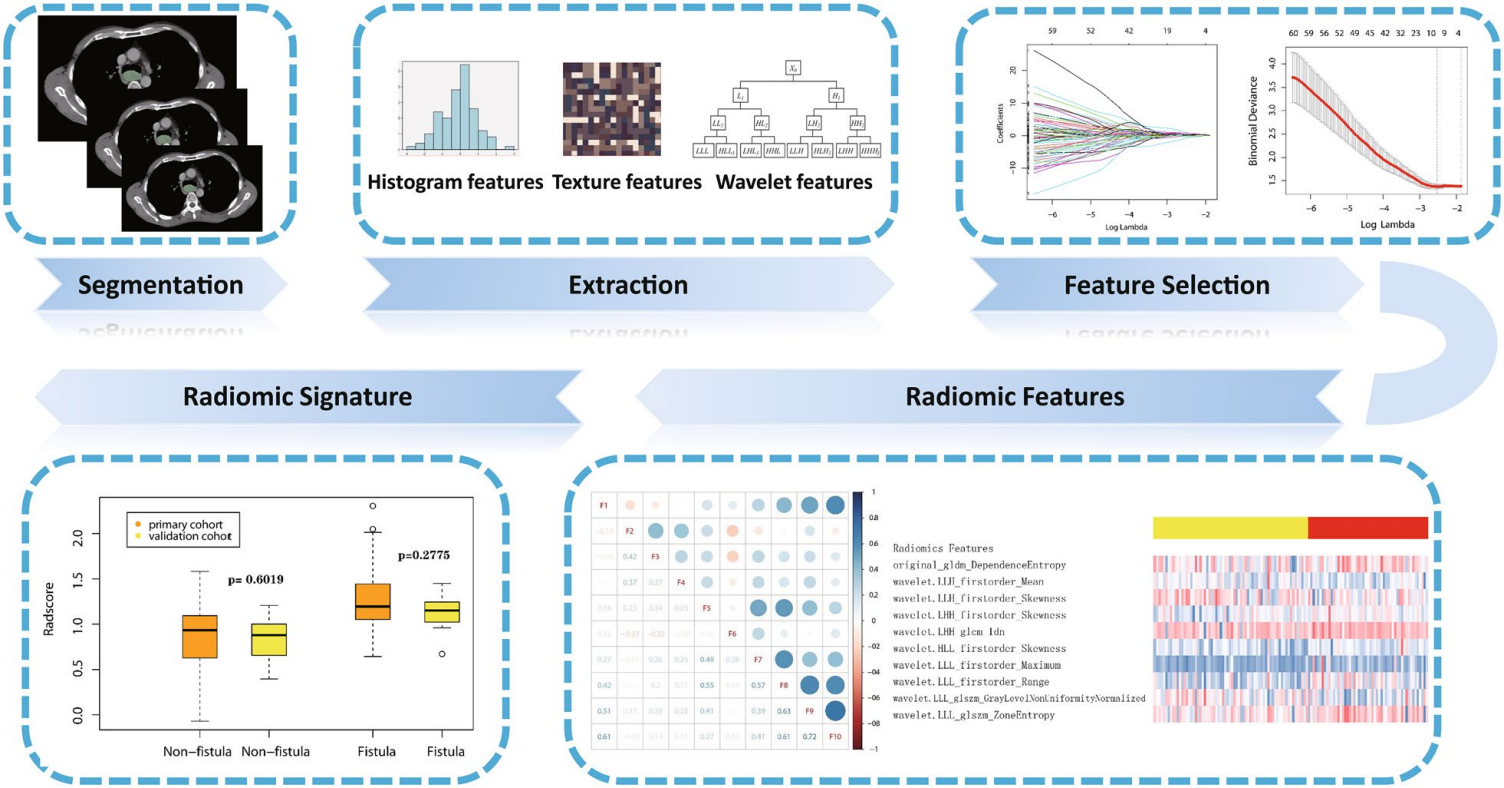
Flowchart of developing a radiomic signature. Following ROI segmentation, 851 radiomic features were extracted from each ROI. The Lasso algorithm selected ten features that had the best correlation with the occurrence of esophageal fistula and a radiomic signature were created. The box chart revealed that there was no significant difference between the training and validation sets, but there was a difference between patients with and without fistula

Two steps were included in radiomics-clinical model construction. First, radiomic signature (radscore) was calculated by adding all filtered eigenvalues multiplied by the corresponding coefficients. The Wilcoxon rank sum (Mann–Whitney) test was applied to the radscores of the training and validation sets to ensure that there was no difference between the two groups. The flowchart for radiomic signature is presented in Fig. 1. Second, the logistic regression algorithm was used to fit radiomic signature and clinical independent risk factors.

### Validation and comparison

In the validation set, the prediction efficiency of each model was evaluated, including discrimination (AUC/ROC), calibration (calibration curve), goodness of fit (AIC), reclassification ability (NRI, IDI), and clinical benefit (DCA curve). The Delong test was used to compare model discrimination abilities.

### Statistical analysis

Stata 15.0 software (Stata Corp, www.stata.com) was used for statistical analysis. The chi-square test was used to compare categorical variables; the Wilcoxon rank sum test was used to compare continuous variables; all tests were two-sided, and *P* < 0.05 was considered statistically significant. R software (version 3.5.3, https://www.r-project.org/) was used to select radiomic features and build models. The R program packages used in this study are listed in the table below.

**Table:** 

Algorithm	Packages	Version
Lasso	glmnet	4.0-1
Logistic, nomogram, calibration	Rms	6.0-1
ROC/AUC	ROCR, pROC	1.0-11, 1.16.2
NRI/IDI	PredictABEL, nricens	1.2-4, 1.6
Correlation test	corrplot	0.84
DCA	Rmda	1.6

## Results

### Patients

This study enrolled 122 patients with esophageal cancer, including 56 patients with MEF. Squamous cell carcinoma was the pathological type of all patients. The time between the occurrence of an esophageal fistula and diagnosis of EC ranged from 1 to 24 months, with an average of 7.87 ± 8.78 months. Esophageal mediastinal fistula occurred in 22 patients, esophageal tracheal fistula in 30 patients, and esophageal pulmonary fistula in 1 patient. Sixty-nine control cases were hospitalized at the same time and had no esophageal fistula within the survival period or 2 years after diagnosis, with a median follow-up time of 30 months (IQR: 16, 38). Table1 shows the baseline characteristics of enrolled patients.

**Table 1:** Clinical characteristics of patients included in the analysis

Characteristics	Primary set		Validation set		χ^2^/*Z*	*P*
	Non-fistula	Fistula	Non-fistula	Fistula		
Subjects	48	38	21	15		
Age (mean ± SD)	63.813 ± 10.097	62.026 ± 9.545	64.0476 ± 6.888	60 ± 7.339	0.478	0.633
Length (mean ± SD)	6.315 ± 2.239	6.297 ± 2.278	5.924 ± 2.497	6.8 ± 2.908	0.328	0.743
Gender						
Female	8 (16.67%)	3 (7.89%)	3 (14.29%)	2 (13.33%)		
Male	40 (83.33%)	35 (92.11%)	19 (90.48%)	13 (86.67%)	0.012	0.913
Stage_T						
T_2_	1 (2.08%)	2 (5.26%)	2 (9.52%)	0 (0.00%)		
T_3_	30 (62.50%)	16 (42.11%)	15 (71.43%)	7 (46.67%)		
T_4_	17 (35.42%)	20 (52.63%)	4 (19.05%)	8 (53.33%)	1.122	0.571
Stage_N						
N_0_	6 (12.50%)	3 (7.89%)	2 (9.52%)	3 (20.00%)		
N_1_	19 (39.58%)	16 (42.11%)	9 (42.86%)	6 (40.00%)		
N_2_	16 (33.33%)	15 (39.47%)	7 (33.33%)	4 (26.67%)		
N_3_	7 (14.58%)	4 (10.53%)	3 (14.29%)	2 (13.33%)	0.511	0.917
Stage_M						
M_1_	15 (31.25%)	12 (31.58%)	10 (47.62%)	8 (53.33%)		
M_0_	33 (68.75%)	26 (68.42%)	11 (52.38%)	7 (46.67%)	3.773	0.052
Location						
Upper	10 (20.83%)	10 (26.32%)	6 (28.57%)	4 (26.67%)		
Middle	17 (35.42%)	15 (39.47%)	10 (47.62%)	6 (40.00%)		
Lower	21 (43.75%)	13 (34.21%)	5 (23.81%)	5 (33.33%)	1.521	0.467
Radiation						
Y	34 (70.83%)	26 (68.42%)	11 (52.38%)	9 (60.00%)		
N	14 (29.17%)	12 (31.58%)	10 (47.62%)	6 (40.00%)	2.271	0.132
Fraction dose						
< 2 GY	14 (29.17%)	13 (34.21%)	5 (23.81%)	3 (20.00%)		
≥ 2 GY	20 (41.67%)	13 (34.21%)	6 (28.57%)	6 (40.00%)	1.671	0.196
Total dose						
< 60 GY	14 (29.17%)	20 (52.63%)	5 (23.81%)	5 (33.33%)		
≥ 60 GY	20 (41.67%)	6 (15.79%)	6 (28.57%)	4 (26.67%)	0.269	0.604
Chemoradiotherapy						
Y	13 (27.08%)	12 (31.58%)	5 (23.81%)	4 (26.67%)		
N	23 (47.92%)	12 (31.58%)	6 (28.57%)	5 (33.33%)	0.068	0.794
Stenosis						
Y	11 (22.92%)	19 (50.00%)	3 (14.29%)	4 (26.67%)		
N	37 (77.08%)	19 (50.00%)	18 (85.71%)	11 (73.33%)	2.863	0.09
Chemotherapy						
Y	45 (93.75%)	34 (89.47%)	21 (100.00%)	14 (93.33%)		
N	3 (6.25%)	4 (10.53%)	0 (0.00%)	1 (6.67%)	1.191	0.275
Chemotherapy regimen^a^						
T^b^	35 (72.92%)	29 (76.32%)	15 (71.43%)	12 (80.00%)		
F^c^	10 (20.83%)	5 (13.16%)	6 (28.57%)	2 (13.33%)	0.266	0.635

^a^The first-line chemotherapy regimen

^b^Paclitexal-based chemotherapy regimen

^c^Fluorouracil-based chemotherapy regimen

In a 7:3 split, all patients were assigned to a training set and a validation set. There were 86 patients in the training set, with 38 (44.2%) having MEF; 36 patients in the validation set, with 15 (41.7%) having MEF. No statistically significant differences existed in baseline clinical characteristics between the two groups (Table 1).

### Clinical predictors and clinical nomogram

**Fig. 2:**
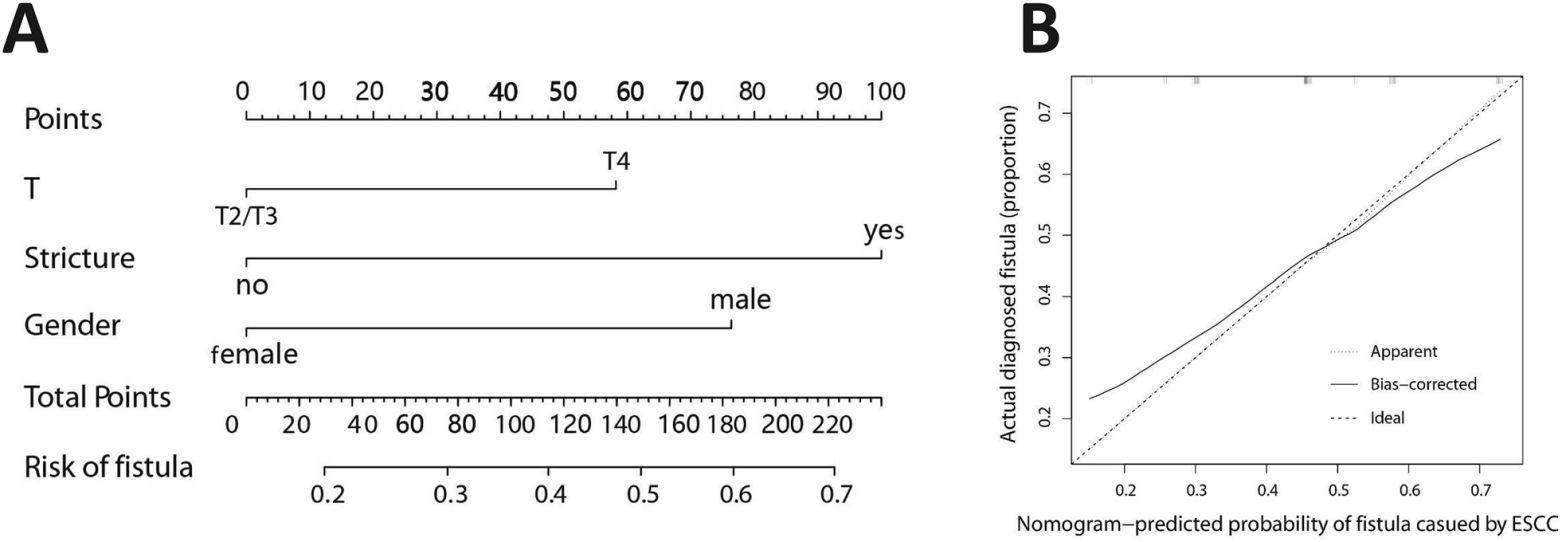
**A** Nomogram developed by clinical risk factors. **B** Calibration curve plotted for clinical prediction model

The clinical prediction model was built using stenosis (*P* = 0.01), gender (*P* = 0.23), and T stage (*P* = 0.11), all of which were screened by logistic regression algorithm (Table 2). The model predicted esophageal fistula with an AUC = 0.691 (95% CI 0.582–0.799) in the training set, and 0.640 (95% CI 0.453–0.827) in the validation set. The AIC value was 115.8. Figure 2 depicts a clinical nomogram and its calibration curve. Each predictor in the nomogram was assigned a score on a point scale. By adding up the total scores projected in the bottom scale, we could estimate the probability of MEF.

**Table 2:** Univariate regression analysis and multivariate regression analysis

Characteristics	Univariate analysis		Multivariate regression	
	OR (95% CI)	*P*	OR (95% CI)	*P*
Age	0.981 (0.938, 1.025)	0.402		
Length	0.997 (0.821, 1.207)	0.972		
Gender				
Female				
Male	2.333 (0.621, 11.280)	0.236	2.698 (5.126, 18.488)	0.267
Stage_T				
T_2_/T_3_				
T_4_	2.796 (0.855,4.897)	0.111	1.429 (5.177, 3.931)	0.486
Stage_N				
N_0_/N_1_				
N_2_/N_3_	1.087 (0.463, 2.558)	0.848		
Stage_M				
M_0_				
M_1_	1.015 (0.401,2.540)	0.974		
Location				
Upper				
Middle	0.882 (0.285, 2.717)	0.826		
Lower	0.619 (0.200, 1.893)	0.400		
Radiotherapy				
No				
Yes	0.892 (0.3530, 2.271)	0.809		
Chemoradiotherapy				
No				
Yes	1.385 (0.490, 3.942)	0.538		
Chemotherapy regimen^a^				
T^b^				
F^c^	0.603 (0.172, 1.902)	0.402		
Fraction				
< 2 GY				
≥ 2 GY	0.700 (0.247, 1.958)	0.497		
Stenosis				
No				
Yes	3.364 (1.355, 8.725)	0.010	3.257 (1.197, 9.374)	0.023
Radscore				
Mean ± SD	2.772 (1.652, 5.144)	0.000	2.709 (1.573, 5.147)	0.001

*OR* odds ratio, *CI* confidence interval

^a^The first-line chemotherapy regimen

^b^Paclitexal-based chemotherapy regimen

^c^Fluorouracil-based chemotherapy regimen

### Radiomic features

A total of 851 radiomic features were extracted from each ROI, and all features can be divided into 4 categories. The ‘shape’ category had 14 features that indicate the shape and size of regions of interest (ROIs) in 2D and 3D spaces. The ‘first order’ represents statistical eigenvalues of voxel intensity, encompassing mean, maximum, and minimum values. Textural features such as Glcm (GrayLevelCooccurenceMatrix), Glrlm (GrayLevelRunLengthMatrix), Glszm (GrayLevelSizeZoneMatrix), Gldm (GrayLevelDependenceMatrix), and Ngtdm (NeighbouringGrayToneDifferenceMatrix) were computed from multiple statistical matrices and characterized the organization Wavelet-based features were defined as first-order and texture features obtained from eight wavelet decompositions of the original CT images. As a result, the total number of radiomic features can be estimated as 14 + (18 + 24 + 16 + 16 + 14 + 5) + (18 + 24 + 16 + 16 + 14 + 5)8 = 851.

Ten features that were closely related to the presence of an MEF were chosen using Lasso-logistic algorithm (Fig. 1). The selected features and their coefficients are shown in Table 3. There was no significant multicollinearity between the features chosen (Pearson correlation coefficients were all less than 0.9).

**Table 3:** Radiomic features selected and their coefficients

Radiomic features	Coefficient
“original_gldm_DependenceEntropy”	0.295
“wavelet.LLH_firstorder_Mean”	− 0.873
“wavelet.LLH_firstorder_Skewness”	− 0.019
“wavelet.LHH_firstorder_Skewness”	0.327
“wavelet.LHH_glcm_Idn”	0.727
“wavelet.HLL_firstorder_Skewness”	1.027
“wavelet.LLL_firstorder_Maximum”	0.750
“wavelet.LLL_firstorder_Range”	0.171
“wavelet.LLL_glszm_GrayLevelNonUniformityNormalized”	− 0.263
“wavelet.LLL_glszm_ZoneEntropy”	0.302

### Radiomics prediction model

The radiomics prediction model was built based on the selected radiomic features to compare with the clinical and radiomics-clinical models. In validation set, the AUC of radiomics model was 0.692 (95% CI 0.516–0.868) and AIC was 111.4.

### Radiomics-clinical nomogram model

The formula for calculating radiomic signature (radscore) was as follows:

$$\begin{aligned} {\text{Radscore}} & = {\text{original}}\_{\text{gldm}}\_{\text{DependenceEntropy}} \times 0.29509193 \\ & \quad + {\text{wavelet}}.{\text{LLH}}\_{\text{firstorder}}\_{\text{Mean}} \times - 0.87347923 \\ & \quad + {\text{wavelet}}.{\text{LLH}}\_{\text{firstorder}}\_{\text{Skewness}} \times - 0.01889205 \\ & \quad + {\text{wavelet}}.{\text{LHH}}\_{\text{firstorder}}\_{\text{Skewness}} \times 0.32706898 \\ & \quad + {\text{wavelet}}.{\text{LHH}}\_{\text{glcm}}\_{\text{Idn}} \times 0.72705274 \\ & \quad + {\text{wavelet}}.{\text{HLL}}\_{\text{firstorder}}\_{\text{Skewnes}} \times 1.02717885 \\ & \quad + {\text{wavelet}}.{\text{LLL}}\_{\text{firstorder}}\_{\text{Maximum}} \times 0.74987525 \\ & \quad + {\text{wavelet}}.{\text{LLL}}\_{\text{firstorder}}\_{\text{Range}} \times 0.17126651 \\ & \quad + {\text{wavelet}}.{\text{LLL}}\_{\text{glszm}}\_{\text{GrayLevelNonUniformityNormalized}} \times - 0.26262914 \\ & \quad + {\text{wavelet}}.{\text{LLL}}\_{\text{glszm}}\_{\text{ZoneEntropy}} \times 0.30172464. \\ \end{aligned}$$

In the training set, patients without MEF had a radscore of 0.873 ± 0.372, while patients with MEF had a radscore of 1.275 ± 0.381. Wilcoxon rank sum test revealed a significant difference (*P* = 0.000). In the validation set, patients with MEF had a radscore of 1.125 ± 0.186, while those without had a radscore of 0.857 ± 0.2187, a significant difference (*P* = 0.000). In the training and validation sets, there was no significant difference between patients with MEF (*P* = 0.278), and those without (*P* = 0.6019). The multivariate logistic regression analysis including radscore, gender, T stage and stenosis revealed that stenosis (*P* = 0.023) and radscore (*P* = 0.001) were the independent risk factors (Table 2).

**Fig. 3:**
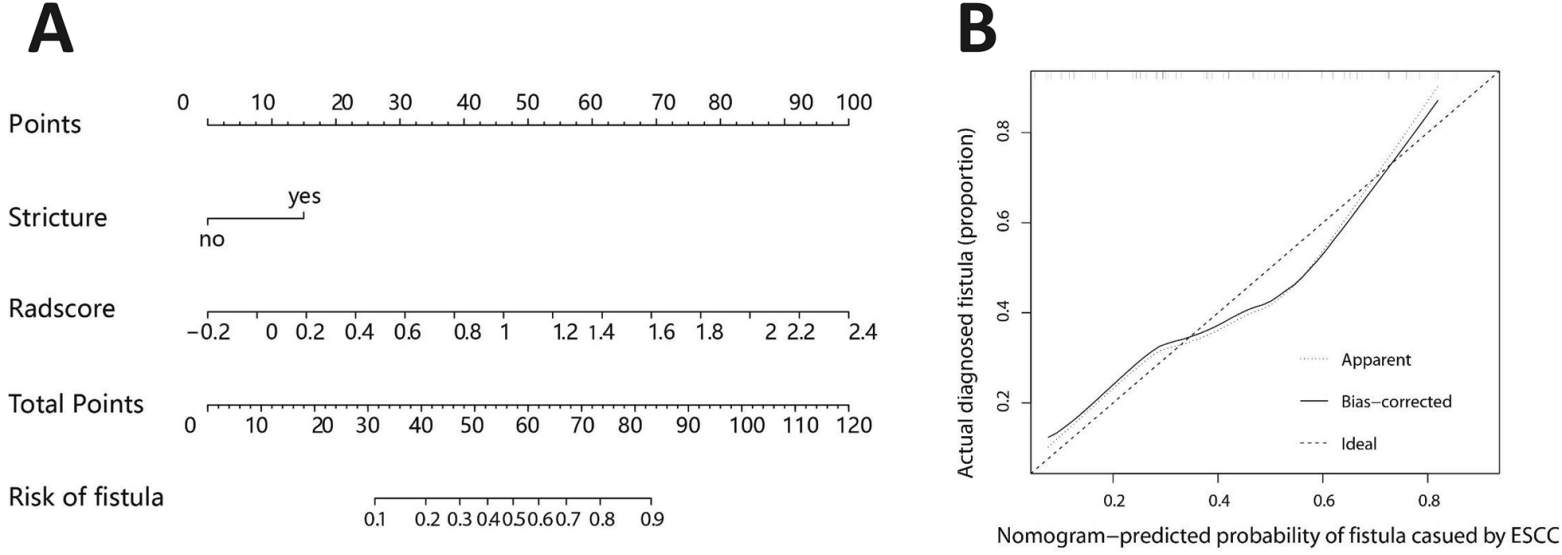
**A** Radiomics-clinical nomogram. **B** Calibration curve plotted for radiomics-clinical prediction model

A radiomics-clinical nomogram prediction model (Fig. 3A) was developed using these two variables as inputs, which predicted esophageal fistula with an AUC = 0.782 (95% CI 0.684–0.8796) in the training set and 0.867 (95% CI 0.7461–0.987) in the validation set, with an AIC = 101.1. The calibration curve revealed that the radiomics-clinical nomogram’s predicted results were in good agreement with the actual observations (Fig. 3B).

### Model comparison

**Fig. 4:**
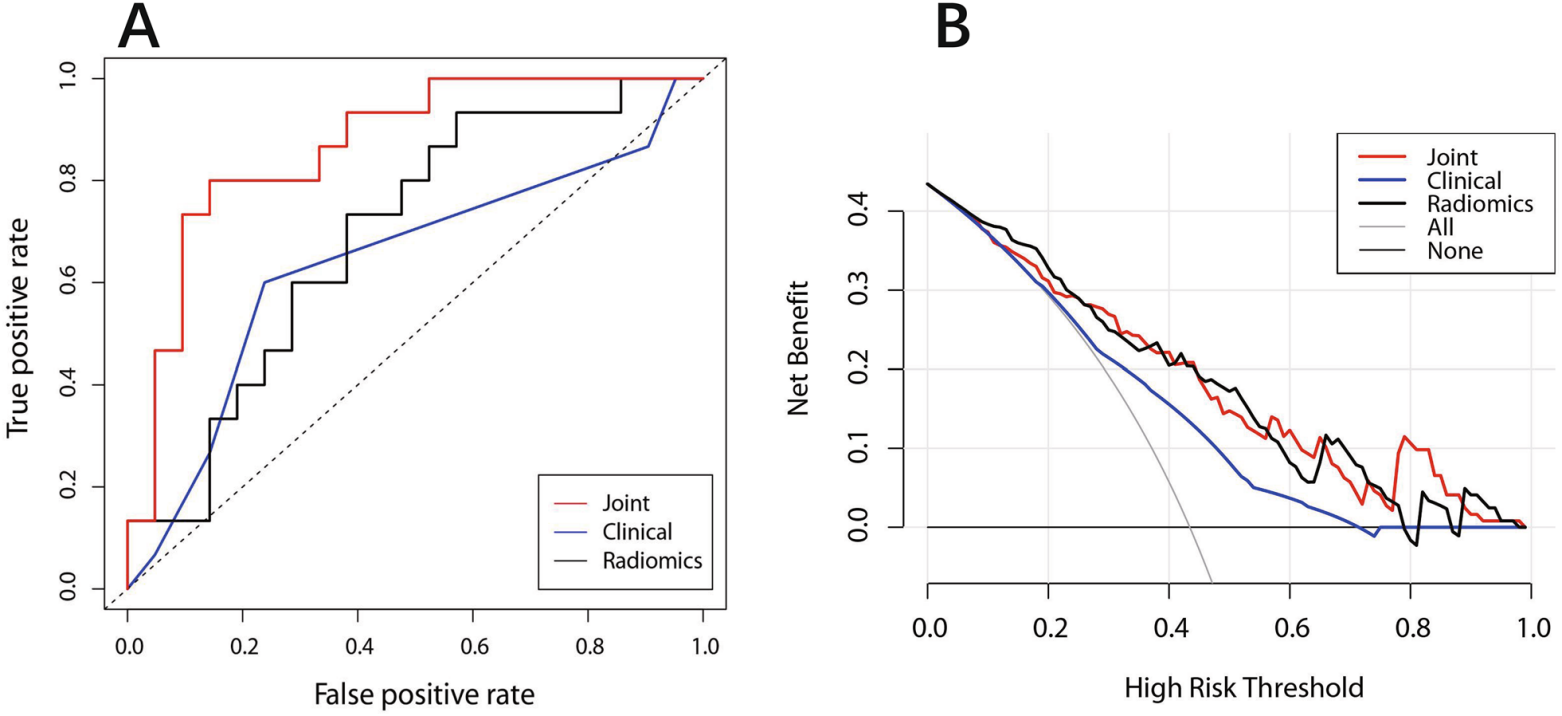
The model’s performance was compared using the **A** ROC curve (discrimination), **B** DCA curve (clinical benefit). Radiomics-clinical prediction model outperformed the other two

Figure 4A displays the discrimination ability (AUC values) of the clinical model, radiomics model, and radiomics-clinical model, revealing that the Joint model outperformed the other two models (Delong test, *P* < 0.05). The net benefit of the radiomics-clinical prediction model under each threshold probability was greater than that of the clinical model and radiomics model, according to the decision curves (Fig. 4B). In comparison to the clinical model, the NRI of radiomics-clinical model was 23.6% (95% CI 0.153–0.614) (1000 iterations), and IDI was 0.125 (95% CI 0.040–0.210), which was statistically significant (*P* = 0.004).

## Discussion

Malignant esophageal fistula is a serious complication of esophageal cancer that significantly reduces the patient’s survival time and quality of life. However, there is currently a scarcity of effective predictive methods. Clinical prediction models and quantitative-CT-based prediction models with AUC of 0.805 and 0.841, respectively, have been reported in previous research [11, 12]. In present study, we built the radiomics-clinical model, clinical model, and radiomics model all at the same time and compared their predictive abilities. We discovered that the radiomics-clinical model performed significantly better than the other two models.

The radiomics-clinical model developed by fitting stenosis with radiomic signature using logistic regression algorithm had an excellent prediction efficiency with an AUC of 0.867 (95% CI 0.7461–0.987). The discrimination ability was significantly higher than that of the clinical (AUC = 0.640) and radiomics models (AUC = 0.692). In terms of goodness of fit, the AIC of the radiomics-clinical model was 101.1, which was lower than the AICs of the clinical model (AIC = 115.8) and the radiomics model (AIC = 111.4), indicating a better fitting performance.

T_4_ indicates that esophageal cancer has infiltrated the entire esophageal layer and has invaded surrounding organs [20]. Esophageal cancer is prone to spontaneous necrosis, and radiotherapy or chemotherapy can also promote necrosis, resulting in an esophageal fistula, particularly in tumors sensitive to radiation or chemotherapy [21]. However, whether radiotherapy is the cause of esophageal fistula remains debatable. Some researchers believe there is no evidence that radiotherapy increases the incidence of esophageal fistula because these patients with tracheoesophageal fistula after radiotherapy may have fistula in any case, but radiotherapy causes it to occur earlier [3]. Other researchers believe that radiotherapy causes tumor tissue to shrink while inhibiting normal tissue repair, which is a major cause of MEF [22, 23].

Esophageal stenosis is another clinically independent predictor. Takahiro et al. [9] discovered that stenosis was the only clinical risk factor for MEF, and that the risk of esophageal fistula in patients with stenosis was twice that of patients without stenosis, which was consistent with our findings. T_4_ and stenosis were also independent risk factors for esophageal fistula in patients treated by concurrent radiotherapy and chemotherapy [24]. However, there is no universally accepted definition of esophageal stenosis, which is usually determined based on the patient’s clinical symptoms and/or the findings of an endoscopic examination [25, 26].

In this study, male patients outnumbered female patients by 6.625:1, and female patients were less likely than male patients to develop an esophageal fistula. Guan et al. discovered that gender was an independent risk factor for malignant esophageal fistula [27]. Previous research has found that gender influenced the prognosis of esophageal cancer [28]. Estrogen regulates metabolism and organ response after injury [29]. As a result, women have natural advantages in injury recovery, which may account for gender differences in the occurrence and prognosis of MEF.

The clinical prediction model’s discrimination was unsatisfactory. To improve model performance, we extracted radiomic features from the primary tumor’s pre-treatment CT, screened out the feature set most relevant to the occurrence of MEF using lasso logistic regression and cross validation, and built a combined model with clinical risk factors, which significantly improved prediction efficiency. The biological behavior of esophageal cancer cells is a significant cause of fistula [30], but clinical risk factors cannot adequately reflect these characteristics. Medical images of tumors are the external manifestation of gene phenotype, and radiomics can convert images into digital data that can be mined, extracted, and analyzed [31]. Radiomics had been used in tumor studies to predict pathological type, stage, curative effect, and prognosis [32–34], and the derived radiogenomics can analyze tumor heterogeneity at the gene level [35, 36]. In our study, the potential 851 candidate radiomics features were eventually reduced to ten potential predictors by the LASSO method for further integration to form the radiomics signature, which contains useful biological information.

Because previous research has shown that arterial phase can better visualize esophageal tumors, which were chosen as the research object in this study [37].

This research had some advantages: first, prior to treatment, all of the clinical characteristics and medical images were gathered, which was helpful in developing the treatment strategy. Second, the nomogram prediction model showed the weight of each parameter on the outcome in a more intuitive way, making it more practical for clinical use. There were some limitations: because none of the patients in this group were surgically treated, only a few patients provided data on tumor differentiation, and this variable could not be assessed. To avoid bias, the total dose of radiotherapy was not included in the analysis. This was due to the fact that eight patients developed esophageal fistulas during radiotherapy, and their irradiation dose was 2–40 Gy. Furthermore, the modeling and validation were all completed in a single center. In future work, we will include data from multiple centers for external validation to improve the model’s generalization.

## Conclusion

We developed and validated the first radiomics-clinical nomogram which could assist clinicians in identifying patients at high risk of malignant esophageal fistula.

## Data Availability

Data and material were available.

## Abbreviations

MEF: Malignant esophageal fistula
EC: Esophageal cancer
CT: Computed tomography
Lasso: Least absolute shrinkage and selection operator
AJCC: American Joint Committee on Cancer
PACS: Picture archiving and communication system
DICOM: Digital imaging and communications in medicine
ROI: Region of interest
ROC: Receiver operating characteristic curve
AUC: Area under curve
AIC: Akaike information criterion
NRI: Net reclassification improvement
IDI: Integrated discrimination improvement
DCA: Decision curve
Glcm: Gray level co-occurrence matrix
Glrlm: Gray level run length matrix
Glszm: Gray level size zone matrix
Gldm: Gray level dependence matrix
Ngtdm: Neighbouring gray tone difference matrix
Glrl: Gray level run length
